# Association of Serum Osteoprotegerin Levels with Bone Loss in Chronic Kidney Disease: Insights from the KNOW-CKD Study

**DOI:** 10.1371/journal.pone.0166792

**Published:** 2016-11-17

**Authors:** Chang Seong Kim, Eun Hui Bae, Seong Kwon Ma, Seung Hyeok Han, Kyu Hun Choi, Joongyub Lee, Dong Wan Chae, Kook-Hwan Oh, Curie Ahn, Soo Wan Kim, Representatives of the KNOW-CKD Investigator Group

**Affiliations:** 1 Department of Internal Medicine, Chonnam National University Medical School, Gwangju, Korea; 2 Depatment of Internal Medicine, Yonsei University College of Medicine, Seoul, Korea; 3 Medical Research Collaborating Center, Seoul National University Hospital and Seoul National University College of Medicine, Seoul, Korea; 4 Department of Internal Medicine, Seoul National University, Seoul, Korea; Tokushima University Graduate School, JAPAN

## Abstract

Osteoprotegerin, a potent osteoclast activation inhibitor, decreases bone resorption and positively affects bone mineral density. This study examined the association between serum osteoprotegerin levels and bone loss in patients with chronic kidney disease, a condition associated with increased risk of mineral and bone disorders. The bone mineral densities of the lumbar spine, total hip, and femur neck were assessed by dual-energy X-ray absorptiometry; serum osteoprotegerin levels were measured at baseline for 1,423 patients enrolled in the prospective KoreaN cohort study for Outcome in patients With Chronic Kidney Disease (KNOW-CKD). Patients aged ≥50 years and with a T-score ≤ –2.5 were diagnosed as having osteoporosis. Multivariable linear regression analysis indicated independent association between serum osteoprotegerin levels and decreased bone mineral density in the lumbar spine (B: –0.489, 95% confidence interval [CI]: –0.883 to –0.095, *P* = 0.015), and total hip (B: –0.349, 95% CI: –0.672 to –0.027, *P* = 0.027). However, bone mineral density of the femur neck was not associated with serum osteoprotegerin levels in women. After adjustments, no independent association was found between serum osteoprotegerin levels and bone mineral density in men. In multivariable logistic regression analysis, serum osteoprotegerin levels were associated with increased risk of osteoporosis in women (odds ratio [OR]: 4.72, 95% CI: 1.35 to 16.52, *P* = 0.015), but not in men (OR: 0.21; 95% CI: 0.04 to 1.31, *P* = 0.095). To summarize, in female patients with chronic kidney disease, increased serum osteoprotegerin levels were independently associated with decreased bone mineral density in the lumbar spine and total hip, and with increased risk of osteoporosis. Therefore, the measurement of serum osteoprotegerin concentration might be useful as a surrogate marker for determining bone loss in patients with chronic kidney disease, especially for women, although not so much for men.

## Introduction

Patients with mild to moderate chronic kidney disease (CKD), or end-stage renal disease have an increased risk for fracture because reduced kidney function is associated with bone loss [1, 2]. The Kidney Disease: Improving Global Outcomes guidelines suggest that bone mineral density (BMD) screening should not be performed routinely for CKD patients due to a lack of association between BMD and fractures in CKD patients with mineral bone disease [3]. However, recent studies showed that low BMD is a risk factor for fracture in patients with predialysis or dialysis CKDs [4–6]. Therefore, assessment of bone loss using BMD may provide information to help anticipate fractures in this high-risk population.

Osteoprotegerin (OPG) is a soluble member of the tumor necrosis factor receptor super family, and a decoy receptor for the receptor activator of nuclear factor-κB (RANK) ligand, which is predominantly expressed by osteoblasts and by the vascular endothelium. OPG plays a critical role in the regulation of bone turnover [7]. OPG specifically inhibits osteoclastic bone resorption and vascular calcification by interfering with binding of the RANK ligand to RANK, as well as promotes the survival of endothelial cells [8–11]. However, a pathological increase of OPG induced inflammation by leukocyte adhesion to endothelial cells [12]. In the clinical setting, a prospective, population-based Bruneck Study showed that OPG was an independent risk factor for the progression of atherosclerosis and for the onset of cardiovascular diseases [13]. Moreover, a cross-sectional study showed that serum OPG levels were positively associated with a high coronary artery calcification score, and could be used as a marker for severe coronary artery calcification in predialysis patients with diabetes [14].

Vascular calcification and bone loss frequently occur together and share same risk factors, such as aging and CKD. Although previous studies showed that serum OPG are associated with vascular calcification, there are limited data regarding the relation between serum OPG levels and bone loss in patients with CKD. A recent retrospective study showed that serum OPG negatively correlated with the BMD of the Ward’s triangle in 31 predialysis patients, but this study population was too small to confirm the results [15]. Therefore, we evaluated the association between serum OPG levels, BMD levels, and osteoporosis in patients with CKD, based on a nationwide CKD cohort study, with further analysis regarding potential gender bias.

## Methods

### Ethics statement

The study protocol was approved by the institutional review board for each of the eight participating clinical centers, including the Seoul National University Hospital, Severance Hospital, Kangbuk Samsung Medical Center, Seoul St. Mary’s Hospital, Gil Hospital, Eulji General Hospital, Chonnam National University Hospital, and Pusan Paik Hospital. All participating patients provided written informed consent. The KoreaN cohort study for Outcome in patients With Chronic Kidney Disease (KNOW-CKD) is supervised by the CKD advisory committee, which comprises individuals from the Korea Centers for Disease Control and Prevention, and from the Korean Society of Nephrology.

### Study design and patient population

KNOW-CKD was launched in 2011, and was a patient-based cohort study that enrolled ethnic Korean adults with CKD. Nephrologists working in clinical centers of the major university-affiliated hospitals, as well as epidemiologists, pathologists, and biostatisticians of a research modulating center are participating in the KNOW-CKD. Data were collected by a well-trained study coordinator using a standardized case report form and protocol. Exclusion criteria included the following: a history of chronic dialysis, organ transplantation, heart failure (New York Heart Association class 3 or 4), liver cirrhosis (Child-Pugh class 2 or 3), history of malignancy, current pregnancy, or single-kidney due to trauma or kidney donation. The detailed design and methods of the KNOW-CKD were published previously [16].

First, we analyzed data recorded between June 2011 and December 2013 for 1,529 KNOW-CKD participants in order to identify the association between serum OPG levels and BMD. Of the 1,529 patients, 106 were excluded from our analysis because their serum OPG levels, BMDs, or T-scores of the lumbar spine, femoral neck, or total hip were not available. A total of 1,423 patients (mean age, 53.4 years; 61.2% men) remained. Second, of the 1,423 patients, those aged under 50 years (n = 497) were excluded from our analysis for osteoporosis, because the target population for our study of osteoporosis prevalence was composed of individuals at higher risk of osteoporosis, namely men aged over 50 years and menopausal women, who are also expected to be aged over 50 years, as the mean age at menopause in Korean women is approximately 50 years [17, 18]. Finally, a total of 926 patients were included in the analysis of osteoporosis prevalence.

### Data collection and definitions

Baseline demographics and laboratory data were retrieved from the electronic data management system (PhactaX) with the assistance of the Division of Data Management of the Seoul National University Medical Research Collaborating Center. Anthropometric measurements including height and weight were conducted at the clinic. Serum samples were collected at baseline according to our standardized protocol, and sent to the central laboratory (Lab Genomics, Korea) for measurement of creatinine, intact parathyroid hormone (iPTH), and 25-OH vitamin D. Other laboratory data were analyzed at the hospital laboratory of each participating center. Serum creatinine concentration was measured using an isotope-dilution mass-spectrometry-traceable method. The definition of CKD stages 1–5 was based on the estimated glomerular filtration rate (eGFR), which was calculated using the four-variable Modification of Diet in Renal Disease (MDRD) formula [19]. The first-voided urine samples were tested for urine creatinine, albumin, and protein concentrations at the central laboratory (Lab Genomics, Korea). Detailed protocols for measurement of other laboratory parameters were previously described [16].

Osteoporosis was defined using the World Health Organization T-score criteria, which states that for the positive diagnosis either the femoral neck or lumbar spine dual-energy X-ray absorptiometry (DXA) scores must be 2.5 or more standard deviations below the sex-specific healthy-young-adult average [20].

### Measurement of serum OPG concentration

Serum OPG (BioVendor R&D) was measured in the central laboratory (Lab Genomics, Korea) by enzyme-linked immunosorbent assay kit. Intra- and inter-assay coefficients of variations were <4.9% and <9.0%, respectively. Samples were assayed in duplicate, and all results were reported as mean values.

### Measurement of bone mineral density

BMD testing was estimated using a Hologic DXA system (QDR4500A Scanner, software version 9.03). BMD was measured for the lumbar spine (L_1_-L_4_), total hip, and femoral neck at baseline. Results were expressed as the T-score (standard deviation from the average BMD value of young normal subject’s).

### Statistical analysis

We examined participant’s characteristics by quartile of OPG concentrations. Serum OPG was skewed, and was therefore natural log-transformed or modeled in quartiles. The demographics and biochemical characteristics were evaluated using the one-way analysis of variance or the Kruskal-Wallis test, and the Pearson chi-square test for continuous variables and categorical variables, respectively. Continuous variables were expressed as mean ± standard deviation or medians with interquartile (25^th^ and 75^th^ percentiles) ranges, and categorical variables were presented as the number and percentage of patients. The relationship between serum OPG levels and each BMD value was assessed using scatter plots. Multivariable linear regression was used to evaluate the association between serum OPG levels and each BMD value at baseline. Moreover, to evaluate the independent association between serum OPG and the risk of osteoporosis, multivariable logistic regression was used. To test whether the association of BMD and the risk of osteoporosis with serum OPG levels differed between male and female CKD patients, analyses were done separately by sex. Significant factors in the univariate analysis were selected as the variables for adjustment. We performed the following analyses: (1) unadjusted; (2) Model 1, adjusted for age and sex; (3) Model 2, adjusted for the variables in Model 1 plus body mass index (BMI); eGFR; log-transformed the urinary albumin-to-creatinine ratio (UACR); diabetes mellitus; hypertension; history of coronary artery disease, cerebrovascular accident, and peripheral vascular disease; current/former smoker; level of total calcium, inorganic phosphate, alkaline phosphatase, serum albumin, intact parathyroid hormone (iPTH), 25-OH vitamin D, and abdominal aortic calcification (AAC) score. Finally, to examine the ability of serum OPG concentrations measures to predict the risk of osteoporosis, we used receiver operating characteristics (ROC) curves. All statistical tests were two-tailed and *P*<0.05 was considered significant. The analyses were performed using the Statistical Package for Social Sciences software, version 21.0 (IBM Corp, Armonk, NY, USA).

## Results

### Baseline characteristics

**Fig 1:**
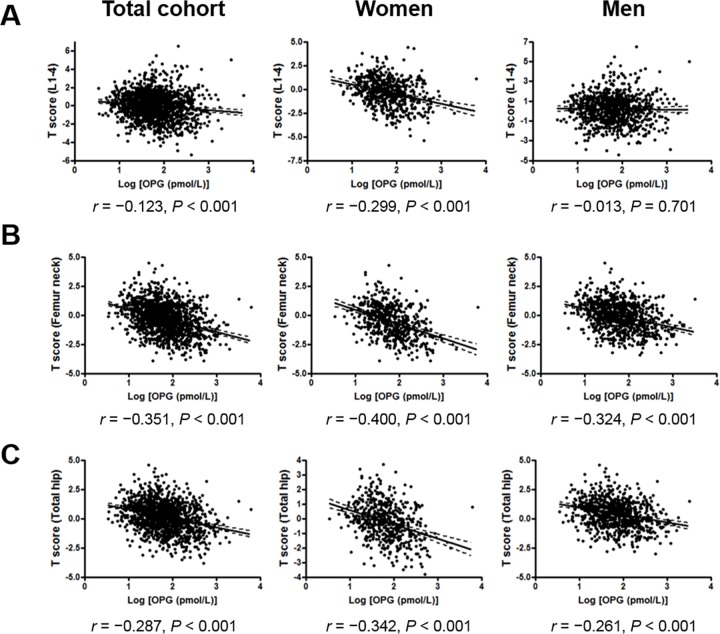
Regression plots between serum osteoprotegerin levels and the bone mineral densities of the (A) lumbar spine, (B) femoral neck, and (C) total hip in chronic kidney disease patients (total cohort, women, men).

The demographic, clinical, and biochemical characteristics of the patients according to quartile concentration are shown in Table 1. The average baseline eGFR was 49.1 ± 29.6 mL/min per 1.73 m^2^, and the median serum OPG concentration was 5.94 pmol/L for the subjects in our study. Compared to patients with serum OPG concentrations in the lowest quartile, patients with higher serum OPG concentrations were older; had a higher prevalence of diabetes, hypertension, coronary artery disease, cerebral vascular accident, and peripheral artery disease; had higher AAC score, inorganic phosphate, total alkaline phosphatase, iPTH, and UACR; and lower total calcium, serum albumin levels, and baseline eGFR. Log-transformed serum OPG concentration correlated with higher CKD stage, and lower eGFR (*r* = −0.484, *P* < 0.001; S1 Fig). Fig 1 shows the regression plots between log-transformed serum OPG concentrations and T-scores for lumbar spine, femoral neck, and total hip BMDs in women and men, respectively. Serum OPG concentration was negatively correlated with lumbar spine (*r* = −0.123, *P* < 0.001), femoral neck (*r* = −0.351, *P* < 0.001), and total hip BMDs (*r* = −0.287, *P* < 0.001) in total cohort. Especially, the negative correlations between serum OPG levels and BMDs of lumbar spine, femoral neck, and total hip are higher for women (*r* = −0.299, *P* < 0.001; *r* = −0.400, *P* < 0.001; *r* = −0.342, *P* < 0.001, respectively), and lower for men.

**Table 1:** Baseline distribution of demographic, clinical, and biochemical characteristics of the study population according to quartile groups of the distribution of values of serum osteoprotegerin levels.

Serum OPG	1st quartile (n = 356)	2nd quartile (n = 355)	3rd quartile (n = 356)	4th quartile (n = 356)	*P* value
Parameters	(≤ 4.39 pmol/L)	(4.40–5.93 pmol/lL)	(5.94–8.17 pmol/L)	(≥ 8.18 pmol/L)	
Age (year)	43.5 ± 10.7	51.4 ± 10.8	56.3 ± 11.0	62.5 ± 8.2	<0.001
Male (%)	230 (64.6)	210 (59.2)	214 (60.1)	217 (61.0)	0.466
Systolic BP (mmHg)	124 ± 15	127 ± 15	129 ± 15	132 ± 19	<0.001
Diastolic BP (mmHg)	77 ± 11	78 ± 11	76 ± 11	76 ± 12	0.070
Current/former Smoker (%)	171 (48.0)	165 (46.5)	169 (47.6)	179 (50.3)	0.145
BMI (kg/m^2^)	24.2 ± 3.4	24.6 ± 3.5	24.5±3.4	24.1 ± 3.0	0.161
Diabetes (%)	35 (10.4)	87 (25.4)	130 (37.4)	222 (62.7)	<0.001
Hypertension (%)	300 (89.6)	328 (95.9)	331 (95.1)	340 (96.0)	<0.001
CAD (%)	5 (1.5)	25 (7.3)	31 (8.9)	49 (13.8)	<0.001
CVA (%)	10 (3.0)	20 (5.8)	32 (9.2)	57 (16.1)	<0.001
PAD (%)	1 (0.3)	0 (0)	8 (2.3)	15 (4.2)	<0.001
AAC score	0.34 ± 1.28	0.83 ± 2.16	1.39 ± 2.77	2.43 ± 3.51	<0.001
Total calcium (mg/dL)	9.23 ± 0.43	9.20 ± 0.45	9.11 ± 0.59	8.92 ± 0.55	<0.001
iP (mg/dL)	3.50 ± 0.58	3.64 ± 0.58	3.73 ± 0.66	3.92 ± 0.75	<0.001
Intact PTH (pg/mL)	50.6 (35.3,69.5)	39.8 (27.0,63.9)	46.1 (31.1,72.8)	55.5 (35.5,91.5)	<0.001
25(OH) vitamin D (pg/mL)	15.6 (10.6,20.7)	16.8 (12.3,22.4)	17.0 (12.7,24.8)	15.8 (11.2,22.4)	0.007
Total ALP (U/L)	64.5 (53.3,93.8)	62.0 (47.0,80.0)	65.0 (51.0,86.0)	71.0 (57.0,93.0)	<0.001
Serum albumin (mg/dL)	4.32 ± 0.33	4.22 ± 0.37	4.17 ± 0.40	4.02 ± 0.43	<0.001
Baseline Cr (mg/dL)	1.35 ± 0.76	1.63 ± 0.95	1.92 ± 1.14	2.57 ± 1.42	<0.001
Baseline eGFR ^a^	68.0 ± 31.8	54.6 ± 29.7	43.8 ± 23.2	30.4 ± 17.5	<0.001
Log-UACR (mg/g)	4.89 ± 1.81	5.31 ± 1.87	5.75 ± 1.90	6.25 ± 1.68	<0.001
VTDR (%)	13 (10.9)	22 (14.8)	15 (9.6)	26 (16.6)	0.243
AVD (%)	3 (2.5)	8 (5.4)	13 (8.3)	17 (10.8)	0.043
CPB (%)	17 (14.3)	42 (26.8)	31 (19.9)	47 (29.9)	0.010

^a^ eGFR is given in ml/min per 1.73m^2^, calculated using the MDRD equation

Abbreviations: OPG, osteoprotegerin; BP, blood pressure; BMI, body mass index; CAD, coronary artery disease; CVA, cerebrovascular accident; PAD, peripheral artery disease; AAC, abdominal aorta calcification; iP, inorganic phosphate; PTH, parathyroid hormone; ALP, alkaline phosphatase; Cr, creatinine; eGFR, estimated glomerular filtration rate; UACR, urinary albumin-creatinine ratio; VTDR, vitamin D replacement; AVD, active vitamin D; CPD, calcium phosphate binders

### Association of serum OPG levels with BMD

We analyzed serum OPG concentration as a continuous variable. The analysis showed that serum OPG levels were associated with BMDs in sequential adjusted models (Table 2). In total cohort, higher serum OPG concentration associated with lower lumbar spine BMD, even after fully adjustment (B, −0.338; 95% CI, −0.599, −0.007; *P* = 0.011). However, femoral neck and total hip BMD did not have association after sequential adjustments. In women, serum OPG concentration were significantly associated with decreased lumbar spine, and total hip BMD, even after sequential adjustments (B, −0.489; 95% CI, −0.883, −0.095; *P* = 0.015; B, −0.349; 95% CI, −0.672, −0.027; *P* = 0.027, respectively). However, serum OPG concentration was not significantly associated with any BMDs in the final multivariable model for men.

**Table 2:** Multivariable linear regression analysis between bone mineral density and log-transformed serum osteoprotegerin levels for patients with chronic kidney disease.

	Total cohort (n = 1423)		Women (n = 552)		Men (n = 871)	
**Log OPG (pmol/L)**	1.78 (1.48,2.10)		1.79 (1.50,2.10)		1.78 (1.46,2.10)	0.426
	B Value (95% CI)	*P* value	B Value (95% CI)	*P* value	B Value (95% CI)	*P* value
**L**_**1**_**-L**_**4**_						
	−0.007 ± 1.480		−0.323 ± 1.491		0.193 ± 1.432	<0.001
Unadjusted	−0.390 (−0.554, −0.226)	<0.001	−1.007 (−1.277, −0.738)	<0.001	−0.039 (−0.238, 0.160)	0.701
Model 1 ^a^	−0.316 (−0.517, −0.116)	0.002	−0.505 (−0.829, −0.181)	0.002	−0.195 (−0.422, 0.052)	0.122
Model 2 ^b^	−0.338 (−0.599, −0.077)	0.011	−0.489 (−0.883, −0.095)	0.015	−0.179 (−0.518, 0.159)	0.298
**Femur neck**						
	−0.245 ± 1.292		−0.528 ± 1.369		−0.066 ± 1.207	<0.001
Unadjusted	−0.975 (−1.111, −0.840)	<0.001	−1.239 (−1.477, −1.022)	<0.001	−0.818 (−0.976, −0.659)	<0.001
Model 1 ^a^	−0.514 (−0.674, −0.353)	<0.001	−0.640 (−0.919, −0.360)	<0.001	−0.437 (−0.629, −0.244)	<0.001
Model 2 ^b^	−0.159 (−0.369, 0.051)	0.137	−0.224 (−0.566, 0.117)	0.198	−0.147 (−0.416, 0.121)	0.282
**Total hip**						
	0.188 ± 1.235		−0.206 ± 1.250		0.440 ± 1.158	<0.001
Unadjusted	−0.759 (−0.893, −0.625)	<0.001	−0.965 (−1.190, −0.741)	<0.001	−0.627 (−0.784, −0.469)	<0.001
Model 1 ^a^	−0.479 (−0.637, −0.320)	<0.001	−0.591 (−0.863, −0.320)	<0.001	−0.410 (−0.604, −0.216)	<0.001
Model 2 ^b^	−0.181 (−0.386, 0.024)	0.083	−0.349 (−0.672, −0.027)	0.027	−0.062 (−0.332, 0.207)	0.649

^a^ Adjusted for age and sex

^b^ Adjusted for age; sex; BMI; eGFR; log-UACR; history of hypertension, diabetes mellitus, cerebrovascular accident, coronary artery disease, and peripheral vascular disease; current/former smoker; levels of total calcium, inorganic phosphate, alkaline phosphatase, serum albumin, intact PTH, and 25-OH vitamin D; and AAC score

Abbreviations: OPG, osteoprotegerin; CI, Confidence Interval

### Association of serum OPG levels with osteoporosis

**Fig 2:**
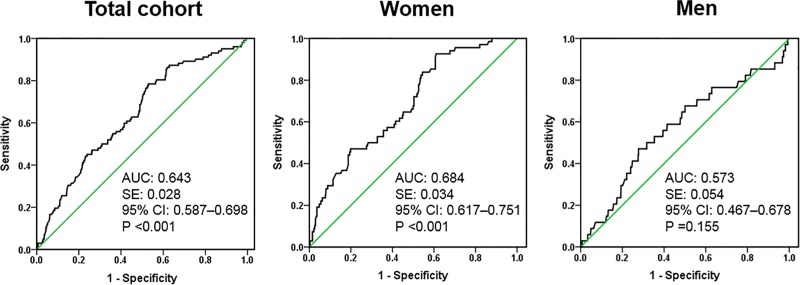
Receiver operating characteristics analysis of the risk of osteoporosis with respect to serum OPG levels in chronic kidney disease patients (total cohort, women, men).

We also performed the multivariable logistic regression analysis to determine the association between serum OPG concentration and the risk of osteoporosis in patients with CKD (Table 3). Serum OPG concentration was associated with an increased risk of osteoporosis for women (odds ratio, 4.719; 95% CI, 1.348–16.520; *P* = 0.015), but these associations were not found for men in either unadjusted or adjusted analyses. The ROC curves analyses for the prevalence of osteoporosis regarding serum OPG concentration in men and women are plotted in Fig 2. The area under the curve (AUC) was 0.684 (95% CI, 0.617–0.751; *P* < 0.001) for women, and 0.573 (95% CI, 0.467–0.678; *P* = 0.155) for men, respectively.

**Table 3:** Multivariable logistic regression analysis of the association between log-transformed serum osteoprotegerin levels and risk of osteoporosis in patients with chronic kidney disease (n = 926, age ≥ 50 years).

	Odds ratio	95% CI	*P* value
**Total cohort (n = 926)**			
Unadjusted	2.902	1.813−4.644	<0.001
Model 1 ^a^	2.305	1.340−3.962	0.003
Model 2 ^b^	1.477	0.614−3.555	0.384
**Women (n = 340)**			
Unadjusted	5.209	2.584−10.503	<0.001
Model 1 ^a^	3.727	1.765−7.871	0.001
Model 2 ^b^	4.719	1.348−16.520	0.015
**Men (n = 586)**			
Unadjusted	1.566	0.726−3.380	0.253
Model 1 ^a^	1.298	0.554−3.043	0.548
Model 2 ^b^	0.213	0.035−1.309	0.095

^a^ Adjusted for age and sex

^b^ Adjusted for age; sex; BMI; eGFR; log-UACR; history of hypertension, diabetes mellitus, cerebrovascular accident, coronary artery disease, and peripheral vascular disease; current/former smoker; levels of total calcium, inorganic phosphate, alkaline phosphatase, serum albumin, intact PTH, and 25-OH vitamin D; and AAC score

Abbreviations: CI, Confidence Interval

## Discussion

The present study investigated the association of serum OPG concentration with BMD and with the risk of osteoporosis in predialysis CKD patients. We showed that serum OPG concentration was significantly correlated with decreased lumbar spine and total hip BMDs in female CKD patients. Moreover, serum OPG concentration was independently associated with increased risk of osteoporosis in both populations. However, there are no independent associations of serum OPG concentration with any BMDs or with the risk of osteoporosis in male CKD patients after adjusting for covariates. These findings suggest that the measurement of serum OPG concentration in patients with CKD might be useful as a surrogate marker for determining the bone loss, especially for women, and not so much for men.

OPG-deficient mice exhibit a decrease in total bone density and medial calcification of the aorta and renal arteries, suggesting that OPG deficiency is associated with osteoporosis and vascular calcification [21]. In addition, transgenic overexpression of OPG in OPG-deficient mice effectively rescues the osteoporotic bone phenotype typically observed in these mice [22]. On the other hand, several experimental studies showed that a pathological increase of OPG levels may contribute to inflammation in the endothelium and ischemic brain, which is characteristic to cardiovascular disease [12, 23]. Recent clinical studies have shown that a high OPG level is associated with cardiovascular disease including acute myocardial infarction and heart failure, vascular calcification, and low BMD in patients with CKD, which is in agreement with our findings [13, 14, 24, 25]. Although it is possible that increased OPG production and release, leading to OPG levels above the physiologic concentration, may decrease bone density and contribute to the vascular pathologic condition, further studies are required to determine the causal relationship between increased OPG levels and lower BMD in CKD patients.

Several studies showed the existence of a relationship between serum OPG levels and BMD in the older population [26–31]. In a cross-sectional study that enrolled 185 postmenopausal women with osteoporosis, the plasma OPG levels were inversely related to spine and femoral neck BMDs even after adjustment, and were shown to contribute to the development of osteoporosis [26]. In addition, serum OPG levels were negatively correlated with lumbar spine and femoral neck BMDs for middle-aged men [30]. However, higher levels of OPG were associated with higher BMDs at the lumbar spine, femoral neck, and total hip for women using estrogen, but not for non-users; and higher levels of OPG were associated with higher BMD at the lumbar spine for men [27]. Nevertheless, other studies found no association between serum OPG and BMD for men or women [32–35]. Although poor renal function is significantly associated with decreased BMD [2], the reason for these conflicting results is unclear. The conflicting results might be related to differences between populations; hormone replacement therapy; or to the fact that most study populations had normal renal function, or did not present available data regarding renal function at baseline.

To date, there are few studies examining the relation between levels of OPG and BMD in patients with CKD or end-stage renal disease, conditions associated with an increased risk of mineral and bone disorders, or bone loss [15, 36]. In a cross-sectional study, 132 patients with predialysis CKD were evaluated based on Z scores of the femoral neck and trochanter. Only the femoral neck Z score was negatively associated with OPG levels [36]. Another cross-section study by Jiang *et al*. examined the association of serum OPG concentration with BMD for 31 participants with predialysis CKD and 16 participants undergoing hemodialysis. In predialysis CKD patients, serum OPG concentration negatively correlated with the BMD of the Ward’s triangle after adjustment, but not with those of the lumbar spine, femoral neck, or trochanter. However, there was no correlation between serum OPG concentration and any BMDs in hemodialysis patients [15]. Although the population enrolled in these cross-sectional studies was small, they showed similar results to our findings regarding the negative association between serum OPG levels and BMDs in patients with predialysis CKD.

However, in our study, only lumbar spine BMD was significantly associated with serum OPG levels for the total cohort, while the BMD at lumbar spine, and total hip were associated with serum OPG levels for women, independent of the abdominal aorta calcification, which may have overestimated the lumbar spine BMD. The reason for discordance in the results regarding the relevant site of BMD in ours and previous studies is unclear. Both cortical and trabecular bones are typically decreased in osteoporosis; however, the mineral bone disease in CKD patients with high PTH levels predominantly decreases the cortical bone, but increases the trabecular bone [3, 37]. Moreover, OPG might affect the cortical and trabecular bones, which may have caused OPG-deficient mice to exhibit a concomitant osteoporotic bone phenotype in an experimental study [7]. However, particular sites, such as the forearm, consist predominantly of cortical bone, while 42% of the vertebral body, and 25% the proximal femur consist of trabecular bone [3]. Taken together, the bone loss configuration at certain skeletal sites, regarding cortical and trabecular bones, might account for the differences, for patient with CKD, in the associations of serum OPG levels with BMDs measured at skeletal sites that have mixed characteristics of bone mineral disease and osteoporosis. Therefore, further studies are needed in order to evaluate the association of serum OPG levels with the cortical and trabecular bone densities in more detail, using high-resolution peripheral quantitative computed tomography.

We found that women had significantly lower BMDs and higher incidence of osteoporosis, especially in women aged over 50 years (data not shown). In our unadjusted analysis, the association between serum OPG levels and BMD was seen for both men and women. However, after adjustment, serum OPG levels were significantly associated with the lumbar spine and total hip BMDs, as well as with the risk of osteoporosis for women. Although several studies indicated a gender bias in the association between serum OPG and BMD in general population, the underlying mechanisms of such gender bias are not well established. Regarding the concentrations of OPG in men and women, a previous study showed that serum OPG levels were higher in women who were using estrogen replacement therapy than in non-user [35]. These results are consistent with *in vitro* studies showing that estrogen administration increased OPG expression, whereas testosterone has an opposite effect [38–42]. Unfortunately, while we did not measure levels of estrogen and testosterone, or record the history of hormonal replacement therapy in our population, we noted no differences in serum OPG concentration between men and women. Hence, we suppose that not only sexual hormones, but also other genetic factors may affect the underlying bone histology and the gender bias, regarding the association of serum OPG levels with BMD and osteoporosis in CKD patients [43].

This study has several limitations. First, despite the fact that the association of serum OPG levels with BMD and with risk of osteoporosis persisted after adjusting for age and other important confounders, we could not rule out the possibility that uncontrolled confounding factors affected our results. Second, this study could not evaluate the levels of estrogen and testosterone, the history of use of oral contraceptives or hormonal replacement therapy, the effects of such hormones on bone turnover, or their biologic interaction with OPG. Thus, the effects of sexual hormones on serum OPG and BMD could not be evaluated. Third, with the progress of the CKD, the underlying bone pathology including cortical or trabecular bone loss naturally becomes more complicated. However, by measuring BMDs using DXA, our study was not able to discriminate between cortical and trabecular bone densities, even though DXA is widely used for measuring osteoporosis in clinical setting [44]. In spite of these limitations, our findings raise the possibility that serum OPG levels may be associated with bone loss, and involved in the risk of osteoporosis for female CKD patients. Moreover, our study included a relatively large population, and determined the gender differences for these associations.

## Conclusion

In predialysis CKD patients, serum OPG levels were independently associated with lumbar spine and total hip BMDs, and with increased risk of osteoporosis for women. However, these associations were not found for men. Additional studies are needed in order to determine whether serum OPG levels represent a cause or an effect of bone loss, and to investigate the therapeutic effect of OPG on bone loss for the predialysis CKD population.

## Supporting Information

S1 Fig (A) Serum osteoprotegerin concentration for each chronic kidney disease stage. (B) Regression plot between estimated glomerular filtration rate and serum osteoprotegerin levels.
(TIF)
