# Supplementary figures and images for: Association of Serum Osteoprotegerin Levels with Bone Loss in Chronic Kidney Disease: Insights from the KNOW-CKD Study
Source: PLoS One. 2016 Nov 17;11(11):e0166792. doi: 10.1371/journal.pone.0166792 (PMC5113973; doi:10.1371/journal.pone.0166792)

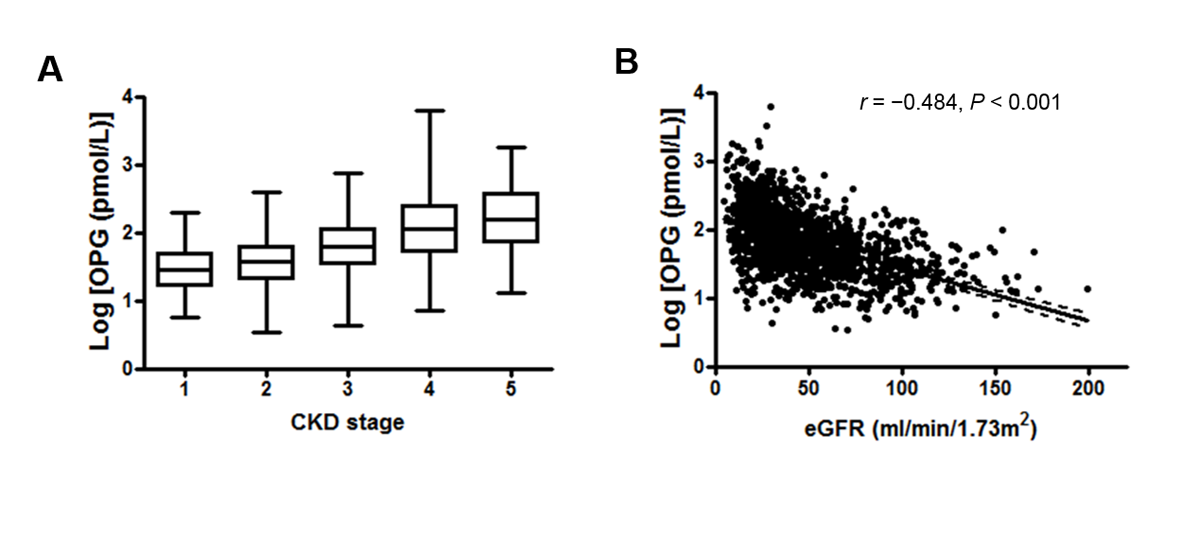

Supplement: S1 Fig — (A) Serum osteoprotegerin concentration for each chronic kidney disease stage. (B) Regression plot between estimated glomerular filtration rate and serum osteoprotegerin levels. (TIF) [file pone.0166792.s001.tif]
